# Phishing URLs Detection Using Sequential and Parallel ML Techniques: Comparative Analysis

**DOI:** 10.3390/s23073467

**Published:** 2023-03-26

**Authors:** Naya Nagy, Malak Aljabri, Afrah Shaahid, Amnah Albin Ahmed, Fatima Alnasser, Linda Almakramy, Manar Alhadab, Shahad Alfaddagh

**Affiliations:** 1 SAUDI ARAMCO Cybersecurity Chair, Department of Networks and Communication, College of Computer Science and Information Technology, Imam Abdulrahman Bin Faisal University, P.O. Box 1982, Dammam 31441, Saudi Arabia; nmnagy@iau.edu.sa; 2 Department of Computer Science, College of Computers and Information Systems, Umm Al-Qura University, Makkah 21955, Saudi Arabia; mssjabri@uqu.edu.sa; 3 SAUDI ARAMCO Cybersecurity Chair, Department of Computer Science, College of Computer Science and Information Technology, Imam Abdulrahman Bin Faisal University, P.O. Box 1982, Dammam 31441, Saudi Arabia; 2190005622@iau.edu.sa (A.A.A.); 2190003750@iau.edu.sa (F.A.); 2190004561@iau.edu.sa (L.A.); 2190001547@iau.edu.sa (M.A.); 2190002162@iau.edu.sa (S.A.)

**Keywords:** parallel processing, cyber-attacks, phishing attacks, machine learning, deep learning

## Abstract

In today’s digitalized era, the world wide web services are a vital aspect of each individual’s daily life and are accessible to the users via uniform resource locators (URLs). Cybercriminals constantly adapt to new security technologies and use URLs to exploit vulnerabilities for illicit benefits such as stealing users’ personal and sensitive data, which can lead to financial loss, discredit, ransomware, or the spread of malicious infections and catastrophic cyber-attacks such as phishing attacks. Phishing attacks are being recognized as the leading source of data breaches and the most prevalent deceitful scam of cyber-attacks. Artificial intelligence (AI)-based techniques such as machine learning (ML) and deep learning (DL) have proven to be infallible in detecting phishing attacks. Nevertheless, sequential ML can be time intensive and not highly efficient in real-time detection. It can also be incapable of handling vast amounts of data. However, utilizing parallel computing techniques in ML can help build precise, robust, and effective models for detecting phishing attacks with less computation time. Therefore, in this proposed study, we utilized various multiprocessing and multithreading techniques in Python to train ML and DL models. The dataset used comprised 54 K records for training and 12 K for testing. Five experiments were carried out, the first one based on sequential execution followed by the next four based on parallel execution techniques (threading using Python parallel backend, threading using Python parallel backend and number of jobs, threading manually, and multiprocessing using Python parallel backend). Four models, namely, random forest (RF), naïve bayes (NB), convolutional neural network (CNN), and long short-term memory (LSTM) were deployed to carry out the experiments. Overall, the experiments yielded excellent results and speedup. Lastly, to consolidate, a comprehensive comparative analysis was performed.

## 1. Introduction

Due to rapidly developing technology, the internet has become a crucial part of our daily lives. In response to the surge in internet applications, several recent attempts have been made to break into computer systems, networks, and devices. Nevertheless, users are observing a rise in the diversity of vulnerabilities and cyber-attack risks. Phishing is one of the possible attacks. Phishing is widely recognized as a leading source of data breaches and the most prevalent deceitful scam of cyber-attacks performed by cybercriminals [1]. According to reports, 611,877 unique phishing sites were detected worldwide from the third quarter of 2013 to the first quarter of 2021 [2]. Phishing attacks have become a significant hazard where the attacker impersonates a trusted entity and sends a fraudulent (spoofed, fake, or otherwise deceptive) message to steal sensitive and valuable data, cause financial loss, cause reputational damage, install ransomware, or deploy other malware infections [3,4]. The spam emails and messages used as weapons contain uniform resource locator (URL) links, which then host uninvited content that directs users to fraudulent websites.

Due to the wide range of techniques used by phishers to carry out attacks and go beyond the current anti-phishing tools, researchers find it very challenging to detect and combat phishing assaults. To detect and nullify the phishing cyber threat, numerous anti-phishing solutions and techniques are constantly being explored and applied, including blacklists, whitelists, pattern-matching filters, visual similarity, and rules. [3].

Moreover, intelligent techniques such as ML and DL are rapidly gaining popularity in the cybersecurity domain and being extensively applied since the previous decade. These techniques owe their success to their capability to learn from available data and thereby extract valuable insights and accurately predict future cases [5,6,7,8,9]. Subsequently, ML can be used to predict whether the websites are phishing or legitimate, including zero-hour websites [10]. An attack that exploits a previously unidentified vulnerability in a computer application that developers have not had the opportunity to address, and patch is known as a zero-day, zero-hour, or day-zero threat. Features of the existing phishing websites are learned and then utilized to predict new phishing features. Inspired by the mentioned successes of ML algorithms, the overarching research question that we strive to answer is: to what extent an ML-based model can successfully detect a phishing website? Unfortunately, ML models require high computational complexity, which usually results in longer computation time. Traditionally, in single processor environments algorithmic bottlenecks cause significant delays in model processing, from training to classification to distance and error calculation and beyond. Additionally, tasks such as cross-validation or parametric search are time intensive. As well, sequential ML computing is often inadequate for large-scale problems, which include real-time tasks or complex simulations. This is the point when parallel computing (PC) offers exceptional opportunities. Lately, parallel and distributed computing has emerged in the recent advances of many noteworthy research disciplines including ML and cybersecurity, resulting in numerous groundbreaking discoveries.

In ML models, in terms of parallelizing computations tasks, tasks without dependencies can be easily parallelized. For instance, once the trained model is stored in memory, several rows can be predicted simultaneously using k-folds cross-validation and hyperparameter tuning with grid search. Moreover, parallelizing the various tasks can occur within each algorithm as well. For instance, in RF, its N trees can be trained simultaneously. The repetitive task of deciding which variable to split on can be parallelized in gradient boosting (GB). However, parallelizing may not be easy for some algorithms, such as derivative-based iterative algorithms, stochastic gradient descent (SGD), or optimization. In DL models, one can think of multiple approaches to parallelize and/or distribute computation across multiple machines and multiple cores. The two commonly known methods to attain faster training time include local training and distributed training. In local training, the model and entire dataset are fitted into the memory of a single machine with several cores. In distributed training, data parallelism or model parallelism can be implemented. Data are dispersed across various machines in data parallelism. This can be used to speed up training or in cases when the amount of data is too enormous to store on a single system. Model parallelism is applied if the model is too vast to be placed into a single machine; it can be split across numerous machines. For instance, a single layer can fit in the memory of a single machine, and serial output connections between machines are included in forward and backward propagation. Model parallelism is not often utilized to speed up the training process; rather, it is employed when a model cannot fit on a single machine.

With the right data, knowledge of algorithms execution, and ambition, there is no limit to what one can attempt with parallel processing in ML and DL. Certainly, as mentioned above, identifying parallelizable portions of code is the most difficult task. However, multithreading, multiprocessing, and computing clusters can be effectively utilized to reduce the execution/processing time of ML and DL frameworks. Therefore, to address the issues mentioned above, we propose this study which contributes to literature as part of a defense against phishing attack comportment by investigating the effectiveness of parallel computing techniques in ML and DL. Four ML and DL models were used throughout the implementation phase, namely, RF, NB, CNN, and LSTM.

The key contributions of this paper include:

- Enhancing phishing URLs detection by applying parallel processing to ML and DL models using different multiprocessing and multithreading techniques in Python with less computation time.
- Achieving maximum speedup without a trade-off in the performance of the implemented models.
- Carrying out a comprehensive comparison of the results obtained from sequential and parallel ML execution. As per the author’s knowledge, the proposed study is the first study to do this in the domain of phishing attacks detection.
- Making a significant contribution to AI, cybersecurity, and parallel computing literature, especially considering the few studies which have applied parallelism in ML in this respective domain.

This paper is structured as follows: Section 2 discusses the existing studies in the literature that utilized sequential and parallel ML techniques. Section 3 presents the methodology followed to carry out the experiments. Section 4 puts forth the experimental setup. Section 5 showcases the results obtained. Section 6 consolidates the paper by providing a conclusion and future directions.

## 2. Literature Review

Several studies have been published addressing the use of ML and DL-based techniques for phishing attacks detection. This section presents the existing literature by summarizing previous studies. The summaries are organized based on the sequential and parallel implementation.

Most of the studies we reviewed implemented sequential ML algorithms to detect phishing attacks. Firstly, Mausam et al. [11] examined popular ML algorithms’ ability to identify phishing URLs. The dataset was obtained from Kaggle, and the authors implemented a Python program to extract the URLs’ most predictive features of phishing. Further, ten features were extracted from the URLs and fed into three ML algorithms, namely, k-nearest neighbor (KNN), RF, and extreme gradient boost (XGBoost). After training and testing, the evaluation showed the best performance by RF with an accuracy of 96.759%.

Simultaneously, Dutta [12] proposed recurrent neural network–long short-term memory (RNN-LSTM) model to detect legitimate and malicious websites. A dataset of 13, 700 URLs (7900: malicious; 5800: legitimate URLs) was crawled using Alexa and the PhishTank datasets. This framework was developed with the help of Python 3.0 on Windows 10 environment (i7 processor). The suggested method showed the highest accuracy (97.4%) using the Phishtank dataset in 3.45 s and an F1-score (96.4) in 4.62 s.

Using the same data sources as [12], Khan and Rana [13] presented a framework to classify legitimate and phishing URLs based on URL heuristics and third-party based features. The authors utilized a dataset consisting of 3000 websites (2000: phishing; legitimate: 1000) collected from PhishTank and Alexa databases. For training and testing, a 70:30 split was performed. They proposed a deep neural network (DNN) model that showed an accuracy of 99.90% by selecting the best ten features. Limitations of this model includes the inability to accurately classify without the third-party features. In addition, when embedded objects are used instead of texts, the model is unable to perform the detection of malicious phishing websites.

Salahdine et al. [14] proposed a phishing attack detection technique. The authors collected and examined 4000 emails (2000: phishing; 2000: legitimate) targeted at the University of North Dakota’s email service to train, validate, and test the models. They created a dataset of 10 relevant features, 8 of which were gathered from the email content and the remaining from the email header, to model these attacks. Further, the dataset has been cleaned of duplicate emails. The classifiers considered were support vector machine (SVM), artificial neural network (ANN), and logistic regression (LR). A parametric examination was performed on each classifier, and the best findings are reported for evaluation. According to the research findings, an ANN model can accomplish better detection with accuracy of 94.5%.

Nonetheless, Kulkarni et al. [15] classified legitimate and phishing websites based on the URLs. The authors applied ML and DL models such as SVM, ANN, decision tree (DT), and naive Bayesian to extract nine features from each individual URL. A dataset consisting of 1353 websites (548: legitimate; 702: phishing; 103: suspicious) was utilized. A 60:40 split was performed for training and testing. The pruned DT outperformed with accuracy (91.5%), true positive rate (TPR) of 90.97%, and false positive rate (FPR) of 7.81%. The limitations encountered in this study were the small dataset size and the usage of discrete features, which reduced the performance of the models.

Hossain et al. [16] study explored the use of ML techniques to discriminate between phishing and safe websites and evaluated their performance. The authors obtained a public dataset from an online repository from Mendeley. The dataset contained 10,000 websites (5000: phishing; 5000: legitimate) websites. Originally, the dataset consisted of 48 features, then authors applied feature extraction methods including parallel coordinates, Pearson and Shapiro ranking to analyze and visualize the dataset, and principal component analysis (PCA) to reduce its dimensionality. Consequently, 27 lexical features were extracted and fed to five ML models for training and testing, namely, KNN, SVM, DT, LR, and RF. Afterward, their performance was evaluated using precision, recall, F1-score, and AUC. The best performance was achieved using RF with an F1-score of 99% which indicates that both FP and FN rates are at satisfactory levels.

Quite interestingly, Vennam et al. [17] developed an ML-based desktop application to distinguish phishing URL attacks from real URLs using images. They acquired the dataset using a dataset upload module. Further, several ML and DL models were used to train and test the obtained dataset, namely, convolutional neural network-LSTM (CNN-LSTM), CNN-BI LSTM, LR, and XGBoost. In addition, an accuracy graph module plotted the accuracy of the techniques mentioned. The highest accuracy (92%) was achieved using XGBoost.

Additionally, Subasi et al. [18] created an intelligent framework to distinguish between real and phishing websites. A publicly available dataset was employed from UCI ML repository incorporating 29 URL features. Numerous classification methods were examined both individually and ensembled in various combinations to find the best performing detection framework. The AdaBoost with SVM classification model showed the best results with an accuracy of 97.61%, F1-score (97.6%), and AUC (99.6%). For future work, the authors are planning on developing some feature selection methods in the presented model to remove the dependency on the webpage content. Moreover, they are planning on developing a model capable of detecting phishing attacks on mobile devices.

However, some studies presented intelligent systems to detect phishing attacks as well; Subasi et al. [19] used different ML tools, such as ANN, KNN, SVM, C4.5 DT, RF, and rotation forest (RoF), to build an accurate system for phishing website detection using WEKA. The authors obtained the dataset from UCI ML repository and investigated multiple data mining algorithms to detect phishing websites and compare their performances using classification accuracy, the area under the ROC curve (AUROC), and the F1-score. According to the results, RF outperformed the other classification methods by getting the highest accuracy of 97.36%. This work can be enhanced by training the classifiers in parallel, as parallelization techniques can speed up the computational process.

Next, Aliya et al. [20] proposed an intelligent system to detect phishing websites. They focused on implementing DL methods to improve the performance and other metrics of the basic systems, using a CNN-LSTM model for detection instead of an RF algorithm, since CNN-LSTM has a huge capacity and powerful capability to generate optimal feature representation on its own using the raw URLs as their input. The dataset used contained 4000 URLs and a 70:30 split was used for training and testing. The data are then transferred to the CNN network to be trained. Then, analysis is conducted using LSTM. This approach results in a 99.1% accuracy, which shows its effectiveness in practice. Moreover, it can overcome previously observed problems.

Only a few studies implemented ML and DL models in parallel in the quest for high performance accuracy with less computation time in the prediction of phishing attacks. Firstly, Sameen et al. [21] have designed PhishHaven with Python version 3.5.4 on LINUX Ubuntu, which is a collective ML-based detection system that can distinguish between phishing URLs created by humans and by artificial intelligence (AI). They utilized a dataset of 100,000 phishing and normal URLs that contained 16 features. Then, they applied a multi-threading strategy to run ensemble-based ML models in parallel, and the findings demonstrated that PhishHaven outperforms the current lexical-based human-crafted phishing URL detection systems, with an accuracy of 98%.

Next, Alzahrani [22] developed a framework to detect phishing and benign websites. A dataset of 20,000 websites (10,000: legitimate; 10,000: phishing) from the Alexa website and PhishTank database were stored on Amazon public cloud storage S3. The experiment was implemented on Amazon EMR using distributed DL models and various machine cores. Three DL models, namely, CNN, LR, and linear regression, were deployed. The hardware *m4.4xlrage EMR (8 nodes, 64 cores) showed the least training time of 6 min for CNN, 4 min for LR, and 2 min for linear regression. LR outperformed with the highest accuracy (99.97%), recall (98.05%), and precision (99.74%).

In this study, Bountakas and Xenakis [23] proposed HELPED framework to detect phishing emails using ensemble ML algorithms. A diverse dataset was combined from multiple sources together with authors’ mailboxes. It consisted of 35,511 emails (32,051: benign; 3460: phishing). The experiments were carried out in a virtual system using an Intel Xeon 4114 processor running at 2.20 GHz over four cores and 12 GB of RAM (OS-Ubuntu 20.04 64-bit) located in a VMWare ESXi server. DT and KNN were selected as the best algorithms as base learners for the ensemble models. To reduce the complexity of the features and boost performance, the algorithms combined content- and text-based features in separate but parallel operations. The framework showed the best performance with soft voting ensemble learning with an accuracy (99.43%), an F1-score (99.42%), and a low training time of 0.0313 s.

Furthermore, Tajaddodianfar [24] proposed a novel character/word-level DL approach called Texception. It predicts whether a given URL leads to a phishing website after receiving an input of the URL. It differs from traditional approaches since it does not rely on manually created features. The dataset was taken from Microsoft’s anonymized browsing telemetry. According to the authors’ intuition, more convolutional layers with varied filter sizes, rather than more sequential layers with constant filter sizes may be better able to capture text patterns. For this, the model included two parallel paths, one for extracting information at the character level and the other for information at the word level. Texception surpasses a traditional text classification method by raising the TPR by 126.7% while maintaining an extremely low FPR of 0.01% and an accuracy of 99.43%.

Boukhalfa et al. [25] suggested an approach to analyze large network traffic data while applying ML algorithms in parallel to detect hidden attacks with less time consumption. The experiment was conducted in stages using a more advanced version of KDD Cup 99, an NSL-KDD dataset that collects network traffic data from a military environment without redundancy. At each stage, the experiment’s big data cluster’s number of nodes increased, and the processing time of the ML algorithms decreased as the cluster’s size increased. The KNN algorithm achieved the highest accuracy value of 99.9%. For future work, the authors are planning to implement a new intrusion detection system (IDS) using the presented approach.

Lastly, Rajput et al. [26] proposed a collaborative approach using parallel machines and cluster computing for fast isolation of spam and ham emails. The cluster approach can increase computing power by speeding up processing without adding any additional cost. The authors solely used header-based filtering techniques to maintain the users’ privacy. The proposed scheme involves the use of natural language processing (NLP) techniques and ML algorithms to identify the key features of emails and classify them as either spam or ham. The NLP components will automatically extract features from emails, such as word frequencies, punctuation, grammar, and syntax. These features will then be used as inputs for an ML algorithm, such as DNN or DT to classify the emails. The results of the proposed model showed an accuracy of over 90%.

Table 1 below presents a summary and comparison of the sequential [11,12,13,14,15,16,17,18,19,20] and parallel [21,22,23,24,25,26] reviewed studies in the existing literature for phishing attacks detection focusing on various ML and DL techniques, dataset utilized, number and type of samples, number of threads/processors, best-performing technique, and lastly, the highest result obtained in terms of accuracy, respectively.

From the reviewed literature, many observations were made, as follows: All the included studies provided high accuracies (above 90%). However, in terms of speed and efficiency, a common and key limitation for most of the included studies is utilizing sequential execution of ML and DL algorithms for a large-scale problem such as phishing attacks detection. ML and DL execution can be time intensive, and for a such complex and sensitive problem, real-time execution is crucial. Furthermore, most studies, even the ones that applied parallel execution, have not mentioned their experimental setup, i.e., did not mention the number of processors, number of threads, number of cores, etc. This limitation does not allow researchers to sense or measure the speedup and efficiency provided by the parallelized solutions. Lastly, compared to studies with parallel ML-based solutions, studies with sequential ML-based solutions utilized a relatively small number of samples ranging from (1353–13,700) samples. Hence, this indicates that sequential ML-based solutions are incapable of handling data-intensive problems, such as discriminating phishing from legitimate URLs, websites, or emails, where thousands or millions of access requests need to be processed. Based on the literature reviewed, proper implementation of parallel ML-based execution can overcome the mentioned challenges and outperforms sequential execution as the Alzahrani [22] study achieved the highest accuracy obtained of 99.97%, applied parallel execution of the LR algorithm. Moreover, Refs. [12,13] are sequential studies that used the same dataset as the parallel studies [21,22]. However, Refs. [21,22] achieved higher accuracies.

## 3. Methodology

This study is based on the previous work conducted by Aljabri et al. [27]. They have employed ML and DL models, namely, RF, NB, LSTM, and CNN, to detect phishing websites from legitimate using a publicly available dataset, named the Malicious and Benign Webpages dataset, which was produced by Singh and Kumar [28]. The used dataset comprised 1.2 M records for training, and 361 K records for testing, with imbalanced class labels distribution (1.5 M: benign; 35,315: malicious). Therefore, the dataset was under-sampled to balance the class label distribution. After under-sampling, the dataset contained 54 K balanced records for training and 12 K for testing. Furthermore, the dataset contained a set of 11 features of various types, such as content-based, URL lexical-based, and network-based features. Several feature engineering and extraction experiments and techniques were applied. Then, features were selected using correlation analysis, analysis of variance (ANOVA), and chi-square to determine the most discriminative features for phishing attacks detection. Eventually, 15 features were selected to train and test the models. Evaluation of the models’ performance included accuracy, precision, recall, and F1-score metrics.

**Figure 1:**
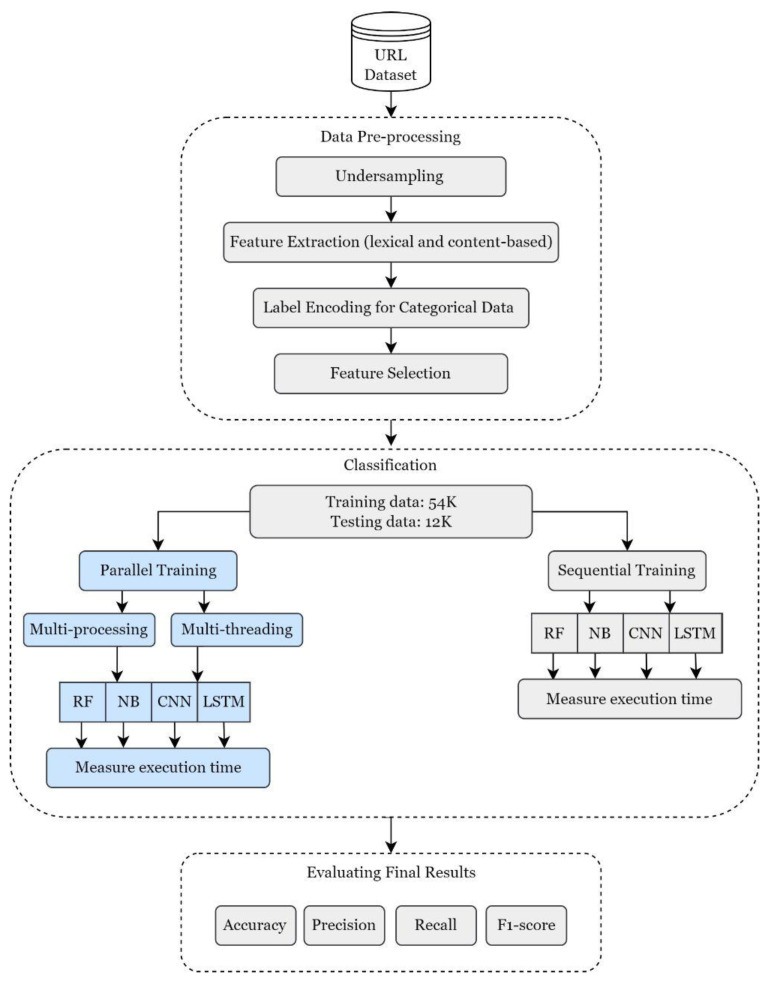
The methodology adopted.

We followed the same methodology adopted by the authors, we used the same dataset, and after they applied the same major steps which are preprocessing, classification, and evaluation. However, in this study, we trained the models in parallel using different multi-processing and multithreading techniques in Python. Then, we measured the training time taken in sequential execution and compared it with the execution time in parallel. Moreover, the models’ performance is re-evaluated after applying the parallel techniques. Lastly, we provided a comparative analysis of the results attained. Figure 1 illustrates a summary of the methodological steps adopted originally in [27] that will be discussed in depth in the following subsections.

### 3.1. Dataset Description

The dataset used is a public dataset called the Malicious and Benign Webpages dataset [28], generated by Singh and Kumar in 2020. Using the Mal Crawler tool [29], they crawled the internet to collect the dataset. Note that the size of the dataset used in this study is one of the largest in the reviewed literature, being outcompeted by [24]. Further, the dataset labels were verified as (good or bad) using Google’s safe browsing API. The dataset includes 11 website features to determine if a web page is malicious or benign, including URL, IP address, JavaScript code, obfuscated code, geographic location, top-level domain, and HTTPS. The full description of the dataset features can be found in Table 2. The dataset was divided into two sets, a training set and a testing set. Table 3 illustrates a summary of number of records per class label in the dataset. As shown in the table the dataset’s class label is highly imbalanced (1.5 M: benign; 35,315: malicious) which is perfectly normal, considering the nature of the problem. From millions of URL accesses, very few will be malicious.

### 3.2. Pre-Processing Phase

The dataset was used after making several pre-processing stages by the researchers in [27].

#### 3.2.1. Under-Sampling

**Figure 2:**
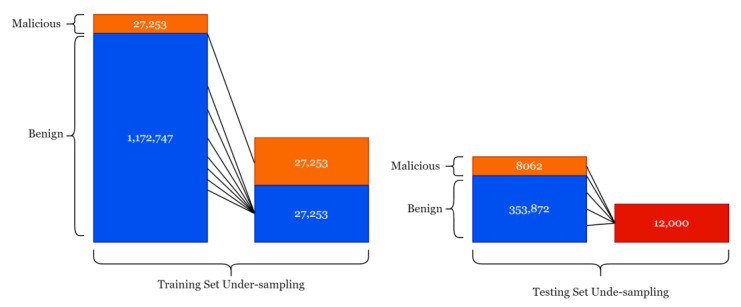
The under-sampling technique for both training and testing sets.

In the pre-processing phase, they used a randomized under-sampling technique that is typically used to balance the class labels in a given dataset by reducing the number of the majority class (benign), in our case to be equal to the minority class (malicious) in our case. The dataset contained 1.2 M records for training: 1.1 M approximately of it was benign, and only 27,253 were malicious. Therefore, it was under-sampled to be equal for each class label (27,253: benign; 27,253: malicious) in the training dataset, thereby making the training set equal to 54,506 records. Moreover, the 361,934 records dedicated for testing were reduced by choosing 12 K records at random; see Figure 2.

#### 3.2.2. Feature Extraction

We used the dataset with the new extracted features by [27]. They were extracted from the existing features (lexical and content-based) in the dataset using many Python and Scikit Learn libraries. The feature extraction process produced 39 new features that are known to be predictive of phishing attacks. In depth feature extraction experiments are out of this paper’s scope. Therefore, for more information refer to [27].

#### 3.2.3. Label Encoding for Categorical Data

After extracting useful features, the categorical data in the dataset were encoded using label encoding, due to the machine’s inability to work well with categorical data, label encoding converts categorical data into numerical form that is understandable to the machine. Label encoding was applied into the class label (0-good, 1-bad), and similarly to HTTPs and who_is features.

#### 3.2.4. Feature Selection

The technique of selecting a subset of the most relevant features in the dataset to the problem is called feature selection. Features selection helps ML and DL algorithms to learn more efficiently and effectively since it uses less memory and reduces time complexity, which is one of the main aims of this study. Feature selection was performed using correlation analysis, ANOVA, and chi-square techniques. For more details, refer to [27]. The feature selection resulted in a final set of 15 feature in the dataset, as listed in Table 4.

### 3.3. Classification Phase

We assessed four ML and DL models, including RF, NB, CNN, and LSTM, in order to train and test the dataset for malicious URL classification. All the models were trained both sequentially and parallelly. In sequential training, the models were trained using only a single thread of execution whereas in parallel training, the models were trained using multiple threads of execution. In this section, we shall further elaborate on the three primary components of our study: sequential training, parallel training, and the evaluation phase.

#### 3.3.1. Sequential Training

The four models—RF, NB, CNN, and LSTM—are trained sequentially in order to obtain the performance metrics and record the execution time. The following sub-sections briefly discuss the four models implemented.

##### Random Forest

RF is an ensemble approach that is mainly used for classification. It advances the common decision tree technique by merging a bigger number of decision trees. RF provides a production based on the most voted class by all trees., i.e., each tree provides a classification or a “vote” then the RF algorithm selects the classification with the majority of votes amongst all trees in the forest. It is known to handle both classification and regression problems [30].

##### Naïve Bayes

NB is a probabilistic model that resembles linear models and is based on the Bayesian theorem. The impact of a feature on a class is presumed to be independent of the values of other features by NB classifiers. The technique is made more efficient while maintaining accuracy due to conditional independence. The model is resilient to parameter changes, performs well on high-dimensional sparse data, and can be used as a baseline [30].

##### Convolutional Neural Network

CNN is a type of DL approach that interchanges weights by leveraging the local connections between surrounding values in both image and sequence data. Convolutional layers in 2D or 3D are widely applied to images. To work with text, however, a 1D convolutional layer was utilized and has been shown to be quite successful, especially when working with time-series or sequence data [31,32]. Because CNN quickly picks up new features, there is less need for manual feature extraction. It is also possible to retrain CNN to carry out new tasks for which it was previously trained [33].

##### Long Short-Term Memory

Long-term dependencies can be learned via LSTMs, a subset of RNNs. Avoiding the issue of long-term dependency is the main focus of the algorithm’s design. LSTMs are structured like chains; however, the repeating module is structured differently [34].

#### 3.3.2. Parallel Training

In the following subsections, the techniques adopted to convert the sequential ML models, namely, RF, NB, CNN, and LSTM, to be trained in parallel are described. The main purpose of this section is to examine, analyze, and explore the possible parallel processing techniques in training ML models.

##### Threading Using Python Parallel Backend

In this technique, the library sklearn joblib must be imported to the working environment to exploit the default parallel backend threading benefits in Python. Using the commands import joblib and from joblib import Parallel, parallel_backend, the ML models in the environment can be trained in parallel, which allows them to take full advantage of the cores available in the machine, and thus speed up the training process. The models can be trained in parallel using the line of code “with parallel_backend(‘threading’):” before fitting the model. Keyword threading is a single-host technique that utilizes the threads to parallelize the execution in the machine.

##### Threading Using Python Parallel Backend and Number of Jobs

This technique uses the same libraries and code that was mentioned above; however, in this technique we add another argument called n_jobs, that allows having more control over the number of working threads. For instance, n_jobs = −1 directs the machine to utilize all the available threads, whereas n_jobs = 1 executes the program in a single thread and so on. It can be used by typing this line “with parallel_backend(‘threading’, n_jobs=#):” before fitting the model.

##### Threading Manually

In this technique, we write our own code to create multiple threads using the threading library to fit the model simultaneously. Nevertheless, this technique is risky as it produces race conditions over the data, especially when training with huge amounts of data.

##### Multi-Processing Using Python Parallel Backend

Similarly, this approach relies on Python’s parallel backend libraries and codes. However, it utilizes processes instead of threads. Mainly, there are two ways to apply this technique, either using “multiprocessing” or “loky” keywords. Both are single-host process-based, but multi-processing is considered as a legacy method. Therefore, loky is preferable.

#### 3.3.3. Evaluation Phase

ML and DL classifiers can be compared in terms of various performance metrics. Accuracy, precision, recall, and F1-score were used in this study to assess and compare the performance of the deployed models. A four-way table with the model’s predicted and actual classifications makes up the confusion matrix, which is used to assess the classifier’s accuracy. Additionally, the sequential and parallel execution time was recorded in seconds and the speedup was calculated.

(i) True positive (TP): correctly predicting benign URLs as benign.
(ii) False positive (FP): incorrectly predicting benign URLs as malicious.
(iii) True negative (TN): correctly predicting malicious URLs as malicious.
(iv) False negative (FN): incorrectly predicting malicious URLs as benign.

Accuracy is defined as the ratio of correctly predicted classes to the total number of instances and is calculated using Equation (1) below:

(1) $$Accuracy=\frac{TP+TN}{TP+FP+TN+FN}$$

Precision measures the proportion of predicted positive classes that are actually positive and is calculated using Equation (2) below:

(2) $$Precision=\frac{TP}{TP+FP}\;$$

Recall is the proportion of correctly predicted classes to all positive classes and is calculated using Equation (3) below:

(3) $$Recall=\frac{TP}{TP+FN}\;$$

F1-score aids in simultaneously measuring recall and precision and is calculated by Equation (4) below:

(4) $$F1\text{-}score=\frac{2\times Precision\times Recall}{Precision+Recall}\;\mathrm{or}\;\frac{TP}{TP+½\left(FP+FN\right)}\;$$

Speedup is the ratio of sequential execution time to parallel execution time and is calculated by Equation (5) below:

(5) $$Speedup=\frac{Sequential\;Execution\;Time}{Parallel\;Execution\;Time}\;$$

## 4. Results and Discussion

### Experimental Setup

To perform the mentioned experiments, we used Python version 3.8.10 on Google Colab notebook platform. The device used has Windows 11 Home operating system. Moreover, the device has core i7 of the eighth generation, which means it works with four cores and eight logical processors. The Malicious and Benign Webpages dataset was used after under-sampling the data, which allowed us to work with 54 K balanced training data, and 12 K testing data. Furthermore, after applying several feature engineering and extraction techniques as discussed in [27], 15 features were selected to train and test the models as shown in Table 4. The detailed parameters’ settings applied for all the models used are shown in Table 5. Five experiments were conducted in total using RF, NB, CNN, and LSTM. The first experiment was based on sequential computing already performed by [27]. The sequential part was reimplemented to be a reference for the given hardware and record the execution time. Followed by the next four experiments based on parallel computing techniques which were elaborately explained in Section 3, namely, threading using Python parallel backend, threading using Python parallel backend and number of jobs with n_jobs = 2, threading manually with two threads, and multi-processing. The results for each model are discussed below in Section 5.

## 5. Results

After applying the parallel techniques mentioned in the methodology section, the accuracy, precision, recall, F1-score, execution time, and speedup were measured for each parallel technique and sequentially. The results for the evaluation metrics were stable and no changes were observed while performing our experiments, which shows that our experiments did not suffer any trade-off between speed and performance. Table 6 below illustrates a summary of the evaluation metrics results obtained for each classifier. All the models provided satisfactory performance. However, NB was the best performing model with an accuracy of 96.01%. Followed by RF and LSTM, and lastly, CNN. For this particular problem, the score of the recall measure is highly significant as well. The recall score shows us how much the model is sensitive to the negative class. In other words, how the model is sensitive to the phishing URLs. One can argue that when an URL is classified as phishing while it is actually legitimate is better than the model classifying the phishing URLs as legitimate. This is exactly the type of information that recall provide us and luckily for the models examined, namely, RF, CNN, and LSTM the recall is 100% which means although their accuracy might not be the highest. However, the sensitivity of the model to phishing URLs is remarkable.

**Figure 3:**
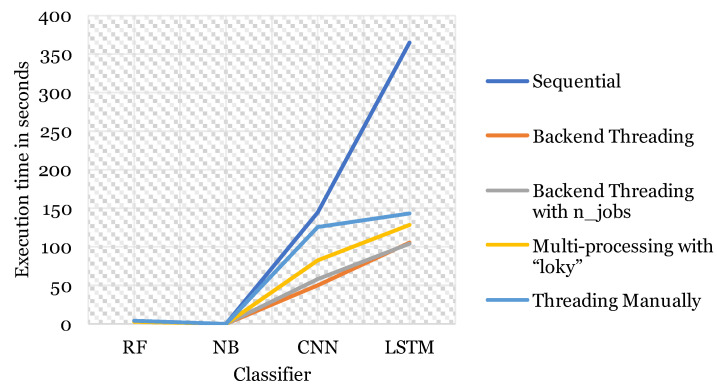
Execution time in sequential comparatively to parallel.

On the other hand, the execution time and speedup varied measurably between each technique and classifier as shown in Table 7. The execution time for ML models sequentially was reasonable as it took NB split-seconds to finish training, which is understandable given that NB is one of the simplest ML algorithms. It works on calculating the probability of each class only, making it one of the fastest ML learners out there. While RF took less than 2 s to finish training. However, the training time for DL models was much longer. It took CNN around 2 min to finish training, whereas LSTM took 6 min of training approximately. In our case, the training time is long but moderate comparatively. Training time can be computationally consuming for the device’s CPU resources, especially in larger datasets, more complex problems, or when using different optimization settings, such as k-fold cross-validation and grid search. Training can take hours to days of processing and power consumption, which is an absurd amount of time, contributing to the issue of overheating. After applying the parallel techniques and measuring the execution time and speedup. Many observations were noted. Interestingly, each classier responded to each technique differently. For instance, RF achieved the best speedup using the backend threading technique with 1.2389× (19.29%) faster training. CNN showed a similar behavior, with 2.9354× (65.93%) speedup using the same technique. Furthermore, LSTM responded very well to the backend threading technique with number of jobs (threads) equal to 2, with a speedup of 3.5134× (71.54%). Lastly, multi-processing resulted in 3.222× (68.96%) speedup for NB classifier. These results indicate that there is no general rule or technique that can be applied to all the classifiers and guarantee the best results possible. The respond of each classifier to each technique varies depending on the nature of those classifiers and the technique itself. However, considering the overall performance of each technique, the backend threading function provided by Python library gave the best results consistently. Followed by backend threading with specifying number of jobs to equal 2, then the multi-processing technique using “loky” keyword, and lastly the least overall speedup was achieved by threading manually. Figure 3 demonstrates these results clearly. Actually, the results attained are sensible. The backend threading performance is better when not specifying the number of jobs or worker threads, because it takes the best advantage of the threads available in the system with no restrictions to a certain number, whereas both threading techniques are superior to the multi-processing technique due to the nature of a process and a thread. A thread is a lightweight process, and therefore, considerably faster. Arguably, when working with threads manually, it produced the least overall results although it is thread-based. In this case, our code produced a race condition between the two threads. Hence, the time taken for communication and synchronization between threads to resolve this condition may have slowed the execution, but it still managed to provide excellent speedup to the sequential execution.

Nevertheless, a possible limitation for this study is that the simulation setup is solid. However, in practical the performance might slightly vary depending on several factors related to the hardware level of the device and processes (tasks) running on the system, such as the load on the machine, number of cores, number of logical processors, memory size, etc. For instance, a machine with heavy load might provide less speedup compared to a machine with light processes running on it. Therefore, the speedup results can be unpredictable which is a common issue in parallel processing, not only in this case. Consequently, the reliability of the results cannot be ensured. However, a speedup is guaranteed even though it might not be significant.

## 6. Conclusions and Future Work

In conclusion, phishing is a deceptive cyber-attack carried out by scammers and hackers to obtain confidential data by impersonating legitimate website. There is a dire need and quest to combat these attacks with high performance and less computation time. In this study, we worked on enhancing sequential phishing URLs detection and classification by applying parallel processing to speed up the training time taken by ML and DL models. For this purpose, we applied multiple experiments in sequential and parallel using multi-processing and multi-threading libraries in Python, firstly by training ML and DL models sequentially, namely, RF, NB, CNN, and LSTM. Then, we evaluated the performance and measured the exaction time for each model. Afterward, parallel experiments using the following techniques, threading using Python parallel backend, threading using Python parallel backend and number of jobs, threading manually, and multi-processing using Python parallel backend. The performance was evaluated again, and the execution time and speedup were measured for each model. Furthermore, the obtained results using sequential and parallel execution were analyzed and discussed in depth. Generally, the sequential execution time of the models was reduced significantly using all the parallel processing techniques with a maximum speedup of 3.5134× (71.54%) achieved for the LSTM model using Python backend threading and the number of jobs set to 2. It is important to note that each classifier responded to each technique differently, which is an indication that there is no such technique that can be applied to all classifiers and guarantee the best speedup possible. However, considering the overall performance, threading using Python parallel backend provided the best results consistently. Remarkably, the speedup of the training time did not affect the evaluation results adversely. Hence, there was no trade-off between performance and speed of execution. The NB model achieved the highest accuracy of 96.01%. However, RF, CNN, and LSTM achieved a recall of 100%. This work results contribute to both the fields of cybersecurity and parallel processing, giving the dearth of research conducted in applying parallel processing to ML solutions. For future work, more research can be conducted to investigate the impact of parallel processing on larger datasets and the usage of graphical processing units (GPUs) on other ML and DL algorithms to speed training time.

## Figures and Tables

**Table 1:** Summary of the reviewed findings.

Ref.	Dataset	No. of Samples	Type of Samples	No. of Threads/ Processors	Best-Performing Technique	Results (Accuracy)
[11]	Kaggle	-	URL	-	RF	96.759%
[12]	Alexa, PhishTank	13,700	URL	-	RNN-LSTM	97.4%
[13]	Alexa, PhishTank	3000	Website	-	DNN	99.9%
[14]	Salahdine-2021	4000	Email	-	ANN	94.5%
[15]	Kulkarni-2019	1353	Website	-	DT	91.5%
[16]	Mendeley repository	10,000	Website	-	RF	F1-score (99%)
[17]	Vennam-2022	10,661	URL images	-	XGBoost	92%
[18]	UCI ML repository	-	URL	-	SVM	97.61%
[19]	UCI ML repository	-	URL	-	RF	97.36%
[20]	Aliya-2021	4000	URL	-	CNN-LSTM	99.1%
[21]	Alexa, PhishTank	100,000	URL	-	PhishHaven	98%
[22]	Alexa, PhishTank	20,000	Website	-	LR	99.97%
[23]	Enron Email Corpus, SpamAssasin Public Corpus, Nazario Phishing Corps, Bountakas-2023	35,511	Email	8 nodes, 64 cores	Soft voting ensemble (DT, KNN)	99.43%
[24]	Microsoft anonymized browsing telemetry	21.7M	URL	-	Texception	99.43%
[25]	NSL-KDD	125,973	Network traffic	5 nodes	KNN	99.9%
[26]	Rajput-2019	9000	Email	4 nodes	KNN	>90%

**Table 2:** The features found in the dataset.

Feature	Description
**url**	Website URL
**ip_add**	Website IP address
**geo_loc**	The geographical location where the website is hosted
**url_len**	Website URL length
**js_len**	JavaScript code length present on the website
**js_obf_len**	The obfuscated JavaScript code length present on the website
**tld**	Website top-level domain
**who_is**	WHO IS domain information is complete or not
**https**	The website is using HTTPS protocol or not
**content**	Raw web page content with JavaScript code
**label**	Class label (malicious or benign)

**Table 3:** The distribution of class labels in the dataset.

	Training Set	Testing Set	Total
**Benign**	1,172,747	353,872	1,526,619
**Malicious**	27,253	8062	35,315
**Total**	1.2M	361,934	

**Table 4:** Final set of features selected.

No.	Features	Description
1	presence_obfuscated_code	Check the presence of obfuscated JavaScript code
2	js_len	Length of JavaScript code present on the website
3	js_obf_len	Length of the obfuscated JavaScript code present on the website
4	count_All_Functions	Count of all the above 7 suspicious functions in content
5	count_find	Count appearance of JavaScript find () function in content
6	count_unescape	Count appearance of JavaScript unescape() function in content
7	count_escape	Count appearance of JavaScript escape () function in content
8	who_is	Who is domain information is complete or no
9	https	Website is using HTTPS protocol
10	count_eval	Count appearance of JavaScript eval () function in content
11	presence_iFrame	the presence of the iFrame tag is checked in content
12	count_search	Count appearance of JavaScript search () function in content
13	presence_Window.open()	the presence of Window.open() function is checked in content
14	host_length	Length of the hostname in URL
15	Count_-	Count “-” symbols in URL

**Table 5:** The parameters’ settings applied for all the classifiers.

Model	Parameter	Value
**RF**	Number of trees	100
**CNN**	Activation function in hidden layers	ReLU
	Number of neurons in output layer	1
	Activation function in output layer	Sigmoid
	Dropout	0.2, 0.5
	Batch size	32
	Number of layers	4
	Number of neurons in hidden layers	32, 64, 64
**LSTM**	Activation function in hidden layers	Tanh
	Number of neurons in output layer	1
	Activation function in output layer	Sigmoid
	Dropout	0.1
	Batch size	32
	Number of layers	3
	Number of neurons in hidden layers	8, 8

**Table 6:** Evaluation results obtained.

	RF	NB	CNN	LSTM
**Accuracy**	95.14%	96.01%	95.13%	95.14%
**Precision**	87.28%	95.65%	87.24%	87.28%
**Recall**	100%	92.25%	100%	100%
**F1-score**	93.21%	93.92%	93.19%	93.21%

**Table 7:** The experiments results summary.

Classifier	Experiments								
	Sequential	Backend Threading		Backend Threading (n_jobs = 2)		Multiprocessing		Threading Manually (2 Threads)	
	Execution Time in (s)	Execution Time in (s)	Speedup	Execution Time in (s)	Speedup	Execution Time in (s)	Speedup	Execution Time in (s)	Speedup
**RF**	1.8246	1.4727	1.2389	1.4848	1.2288	1.6625	1.0975	3.6941	0.4939
**NB**	0.0899	0.0328	2.7408	0.0357	2.5182	0.0279	3.2222	0.07198	1.2503
**CNN**	143.7115	48.9572	2.9354	57.4927	2.4996	82.2356	1.7475	125.6283	1.1439
**LSTM**	364.2227	104.2447	3.4939	103.6654	3.5134	127.1653	2.8641	142.9371	2.5481

## Data Availability

The dataset used in this study is available on request from the authors.
